# Genome Update. Let the consumer beware: *Streptomyces* genome sequence quality

**DOI:** 10.1111/1751-7915.12344

**Published:** 2016-01-06

**Authors:** David J. Studholme

**Affiliations:** ^1^ BiosciencesUniversity of ExeterGeoffrey Pope Building, Stocker RoadExeterEX4 4QDUK

## Abstract

A genome sequence assembly represents a model of a genome. This article explores some tools and methods for assessing the quality of an assembly, using publicly available data for Streptomyces species as the example. There is great variability in quality of assemblies deposited in GenBank. Only in a small minority of these assemblies are the raw data available, enabling full appraisal of the assembly quality.

## Introduction

In a previous Genome Update, we surveyed and discussed some recently published genome sequences of biotechnologically relevant bacteria belonging to the genus *Streptomyces* (Harrison and Studholme, 2014). Despite the undoubted utility of these data resources, a ‘genome sequence’ is actually nothing more than a model or hypothesis. Despite impressive technological advancements in sequencing of relatively long single molecules of DNA, it is still not possible to accurately and unambiguously read the sequence from one end of chromosome through to the other. Rather, we shred the genome into millions of small fragments, incompletely and inaccurately estimate nucleotide sequences for these fragments and then try to piece these together by *de novo* assembly (Nagarajan and Pop, 2013). The result is not a faithful reproduction of the exact primary structure of the target genomic DNA; rather, it is a model, a hypothesis, our best guess at the biological reality. There are choices to be made at each step of the genome sequencing pathway, and each decision may impact on the quality of the final assembly. For example, we previously highlighted differences in two genome assemblies of *Streptomyces* species Mg1 based on data from the Pacific Biosciences SMRT sequencing platform versus data from Illumina plus 454 platforms (Hoefler *et al*., 2013; Harrison and Studholme, 2014). And even starting from the same raw sequence data, it is perfectly possible to generate different final assemblies by choosing different assembly software, choosing different options and parameter values or by different quality‐control filtering regimens (Del Fabbro *et al*., 2013). So, how do we know whether a given genome sequence assembly is a good (or bad) approximation to the biological reality of the real genome? In this Genome Update, I will briefly explore these issues using the bacterial genus *Streptomyces* as an example.

## Length matters: contigs, supercontigs and scaffolds

**Figure 1:**
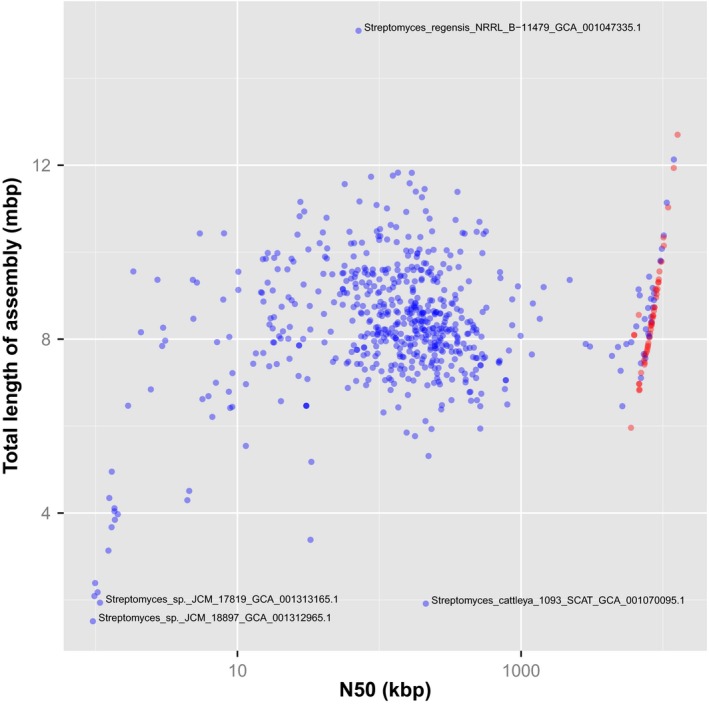
Lengths and levels of contiguity in *Streptomyces* genome assemblies. Total length of the assembly is plotted against the N_50_ length for each of the 653 publicly available *Streptomyces* genome assemblies. The majority of the assemblies consist of multiple contigs or scaffolds and are displayed in blue. The subset of assemblies that are essentially complete and consist of just one or two contigs are displayed in red. Several ‘outlier’ assemblies with very long or very short total lengths are labelled with bacterial strain name and assembly accession number. The total lengths and N_50_ values were calculated using QUAST (Gurevich *et al*., 2013). N_50_ is defined as the length of the shortest contig or scaffold such that 50% of the genome is contained in contigs (or scaffolds) of N_50_ or longer.

The ideal assembled sequence should contain the same number of base pairs as does the real DNA molecule that it represents. Therefore, we should be very suspicious of a sequence assembly whose length is atypical. For example, consider the 653 *Streptomyces* genome assemblies available in GenBank. The median length of these assemblies is 8 330 448 base pairs and most of the assemblies fall within the range of 7–10 megabase pairs (See Fig. 1). However, there are some clear outliers. For example, one assembly exceeds 15 megabase pairs, namely *Streptomyces regensis* NRRL B‐11479 (GCA_001047335.1). A possible explanation for the aberrantly large assembly is that the sequenced material contained more than one organism. This explanation is supported here by the fact that a significant proportion of the predicted proteins share the greatest sequence similarity with *Saccharomonospora* species rather than with *Streptomyces* species.

Similarly, there are several outliers whose lengths are shorter expected. These include assemblies of genomes of *Streptomyces* sp. JCM 18897, *S. cattleya* 1093_SCA, *Streptomyces* sp. JCM 17819, *S. mexicanus* JCM 12681, *S. lavendulae* JCM 4055 and *S. chrestomyceticus* JCM 4735, which are all shorter than 2.5 megabase pairs and so each probably represents less than a third of the actual genome length. These are extreme cases; but they remind us to be cautious about making inferences about genome size from sequence assembly data alone. In a study of genome reduction in *Streptomyces albus*, the authors wisely used pulsed‐field gel electrophoresis to check that the observed restriction pattern matches that predicted by their genome assembly (Zaburannyi *et al*., 2014).

## Completeness

**Figure 2:**
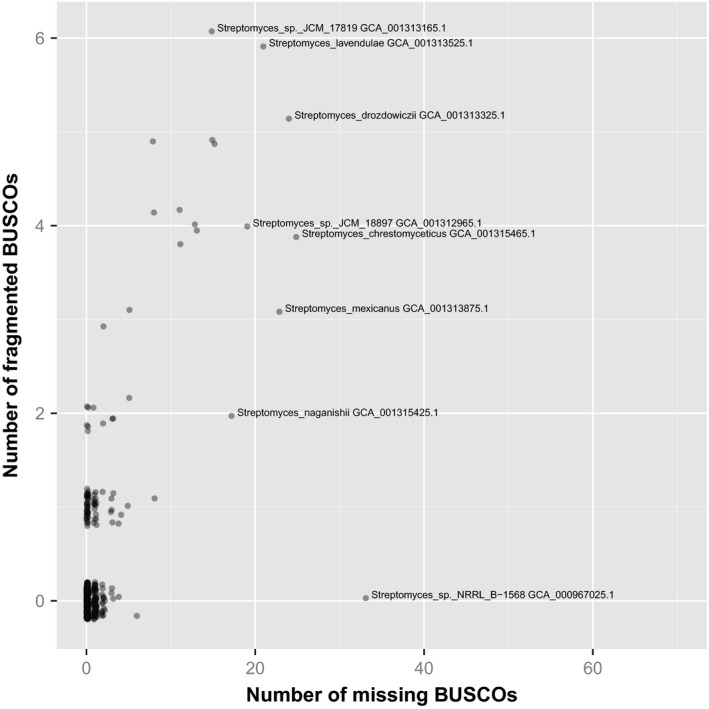
Assessing the completeness of *Streptomyces* genome assemblies. The completeness of each of the 653 publicly available *Streptomyces* genome assemblies was assessed using BUSCO (Simão *et al*., 2015). This tools checks for the presence of each of 40 ‘Benchmarking Universal Single‐Copy Orthologs’ (BUSCOs), which are each expected to be found as a single copy in every bacterial genome. The BUSCO tool checks whether each of these 40 genes is complete or fragmented in the genome assembly. Examples of assemblies with high numbers of fragmented and/or missing BUSCOs are labelled with bacterial strain name and assembly accession number.

If a genome assembly is complete, then it should contain all of the genes, intact. A commonly used tool for assessing completeness of gene‐space in genome assemblies is Core Eukaryotic Genes Mapping Approach (CEGMA) (Parra *et al*., 2007, 2009). However, the software is, as of 2015, no longer actively developed and supported and the authors point out that the underlying set of orthologues that CEGMA uses is based on a database more than a decade old. A more recently published tool, Benchmarking Universal Single‐Copy Orthologs (BUSCO), has a number of advantages (Simão *et al*., 2015) and effectively replaces CEGMA. Figure 2 summarizes the results of running BUSCO against all 653 publicly available *Streptomyces* genome assemblies. This analysis checks for sequences encoding each of a set of 40 BUSCOs that are conserved across all bacteria and usually occur as a single copy per genome. If any of these are missing, then the genome assembly is almost certainly incomplete. Only 63% of genome assemblies (414 of 653) had none of the BUSCOs missing nor fragmented, implying that more than one in three assemblies are, to some extent, incomplete. Not surprisingly, some of the most incomplete assemblies as judged by BUSCO (Fig. 2) were also the shortest and most fragmented assemblies (Fig. 1), such as strains JCM 178919 and JCM 18897. The median number of BUSCOs that occurred complete in duplicate copies was one per genome assembly. However, in the assembly of *S. regensis* NRRL B‐11479, the majority (36 of 40) BUSCOs were duplicated, further supporting the contamination of this data set with sequence from another organism.

## Accuracy

Correctness or accuracy of a genome assembly does not necessarily correlate with contiguity, length or completeness (Magoc *et al*., 2013). So how can we identify errors in an assembly? If the genome being assembled has a reference sequence available, then it is possible to compare the new assembly against the reference and any discrepancies can be reported as errors. The quast software (Gurevich *et al*., 2013) provides a straightforward tool for performing such a comparison and can enumerate structural variations, such as rearrangements, insertions, deletions, different repeat copy numbers, *etc*., as well as single‐nucleotide substitutions. However, most *de novo* genome assembly projects are targeting genomes that have not been previously sequenced and so there is no available reference sequence to provide a ‘ground‐truth’. A closely related genome could be used as reference but in that case it is not readily possible to distinguish misassemblies from true biological variations. Therefore, a reference‐free approach is required.

**Figure 3:**
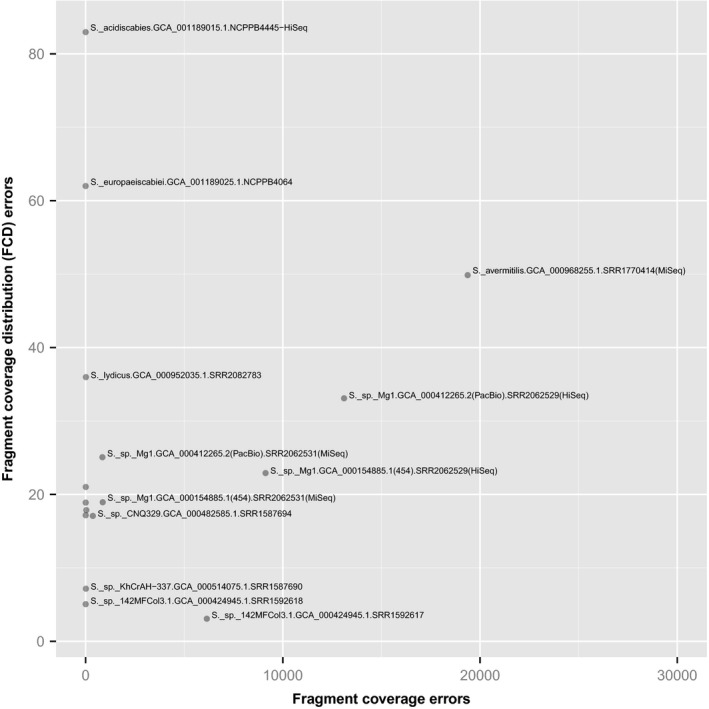
Identification of potential assembly errors in *Streptomyces* genome assemblies. The REAPR tool (Hunt *et al*., 2013) was used to enumerate potential errors in each of the 11 publicly available *Streptomyces* genome assemblies for which Illumina HiSeq reads were available in the Sequence Read Archive (SRA). REAPR aligns sequence reads against a genome assembly allowing detection of anomalies in coverage. It flags two classes of potential errors: fragment coverage distribution (FCD) errors and low fragment coverage errors. We plotted the frequencies of these two classes of potential error for each of the genome assemblies. Example assemblies with very high or very low rates of error are labelled with the name of the bacterial strain, assembly accession number and the SRA accession number.

Reference‐free approaches to verification examine the positions of sequence read pairs within an assembly to identify anomalies that suggest assembly errors. An example of such an approach is Recognition of Errors in Assemblies using Paired Reads (REAPR), described in a paper that also provides an excellent review of the general approach (Hunt *et al*., 2013). REAPR aligns sequence read pairs against a genome assembly (using SMALT http://sourceforge.net/projects/smalt/files/) and analyses the resulting alignments, looking for anomalies in patterns of coverage of the assembly by reads. Essentially, it is looking for deviations from smooth, near‐uniform coverage of the assembly by aligned read pairs. It flags two classes of potential errors: fragment coverage distribution errors and low fragment coverage errors. We plotted the frequencies of these two classes of potential error for each of the genome assemblies where Illumina HiSeq read pairs were available (Fig. 3).

**Figure 4:**
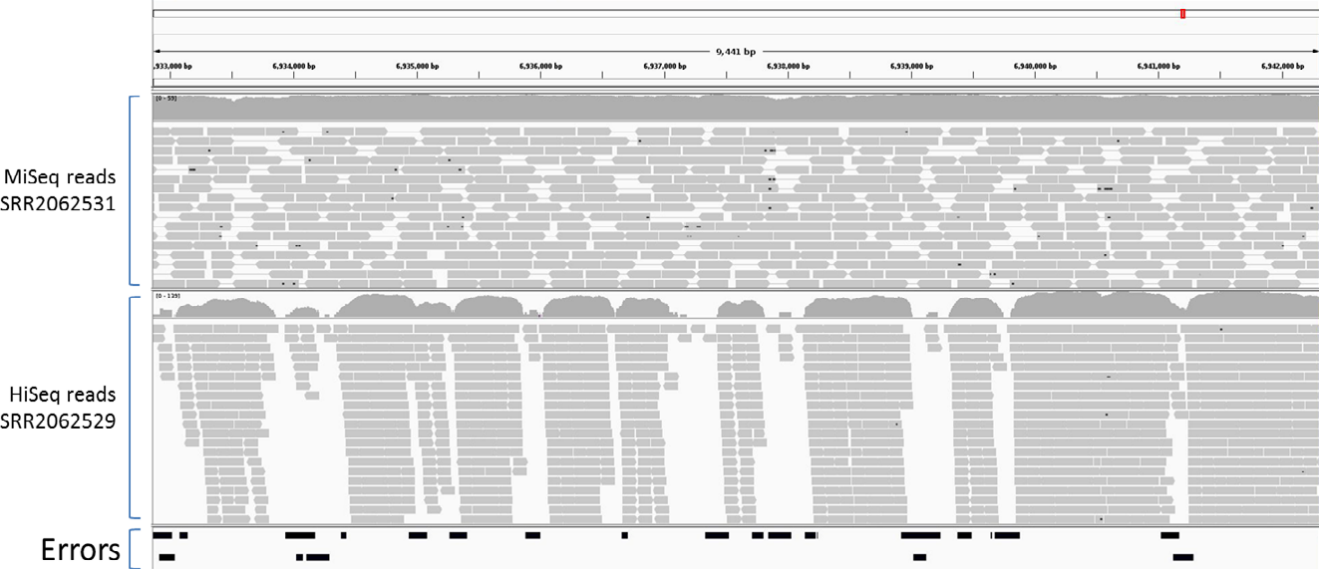
Potential errors flagged by REAPR in the genome assembly of *Streptomyces* sp. Mg1. Shown is a 9.4 kilobase pair region of the genome assembly (assembly accession number GCA_000154885.1) against which Illumina HiSeq and MiSeq reads have been aligned by the REAPR pipeline, which uses SMALT as its alignment engine (Hunt *et al*., 2013). Positions of assembly errors flagged by REAPR are indicated by the black bars.

Clearly there is some variation in the frequencies of assembly errors detected by REAPR. Take for example, two different assemblies of strain Mg1 discussed in a previous Genome Update (Hoefler *et al*., 2013; Harrison and Studholme, 2014). When judged by REAPR using sequence reads from the same Sequence Read Archive (SRA) accession SRR2062529, the assembly based on Pacific Biosciences long‐read sequencing technology appears to have a high number of errors (see assembly GCA_000412265.2 in Fig. 3). However, on closer inspection of the data, we can see that this ‘error’‐rate is more a reflection of the quality of the Illumina HiSeq data in SRR2062529 than the assembly itself; Fig. 4 illustrates a representative segment of the assembly against which are aligned the HiSeq data from SRR2062529 and also another data set from the same strain (SRR2062531 generated using Illumina MiSeq). The REAPR ‘errors’ coincide with gaps in coverage by SRR2062529. On the other hand, the coverage by SRR2062531 is much more smooth and uniform, suggesting good agreement between the raw data and the genome assembly. Figure 3 provides further illustration that the number of ‘errors’ is not only determined solely by the assembly but also by the read pairs used to assess the assembly. Note that REAPR reports different numbers of errors for assembly GCA_000424945.1 depending on which set of sequence reads is used (SRR1592617 or SRR1592618). This likely reflects the different sizes of the two data sets (1.4 and 6.4 gigabase pairs respectively). When assessed using the larger data set, it appears that assembly GCA_000424945.1 is near‐perfect with very few potential errors.

## Conclusion

Clearly not all *Streptomyces* genome assemblies are of consistent quality, whether that quality is measured as size, contiguity, completeness or rate of misassembly. There is a lot of garbage in the databases as well as some very high‐quality data. It is probably reasonable to extrapolate this observation to published genome sequences of all other bacterial taxa and beyond. With the availability of excellent user‐friendly tools such as QUAST, BUSCO and REAPR (among others), there is no excuse not to make some assessment of the quality of a genome assembly prior to publication or indeed afterwards. Unfortunately, there is a tendency for genome authors to fail to deposit the raw sequence data in an appropriate public repository such as the SRA (Leinonen *et al*., 2011), meaning that for the majority of genome projects, the validity of the published assembly can neither be validated by peer reviewers and readers nor independently replicated. *Caveat emptor, quia ignorare non debuit quod jus alienum emit*.

## Funding information

No funding information provided.

## Conflict of interest

None declared.
